# Health-related quality of life and supportive care needs in young adult cancer survivors—a longitudinal population-based study

**DOI:** 10.1007/s00520-024-08896-3

**Published:** 2024-10-22

**Authors:** Alexandra Wide, Johan Ahlgren, Karin E. Smedby, Kristina Hellman, Roger Henriksson, Olof Ståhl, Claudia Lampic, Lena Wettergren

**Affiliations:** 1 Department of Public Health and Caring Sciences, Uppsala University, BMC, Box 564, 751 22 Uppsala, Sweden; 2 Department of Oncology, Faculty of Medicine and Health, Örebro University, Örebro, Sweden; 3 Regional Cancer Centre Mellansverige, Uppsala, Sweden; 4 Department of Medicine Solna, Clinical Epidemiology Division, Karolinska Institutet, Stockholm, Sweden; 5 Department of Hematology, Karolinska University Hospital, Stockholm, Sweden; 6 Department of Gynecologic Cancer, Theme Cancer, Karolinska University Hospital, Stockholm, Sweden; 7 Department of Radiation Science and Oncology, Umeå University, Umeå, Sweden; 8 Department of Oncology, Skåne University Hospital, Lund, Sweden; 9 Department of Psychology, Umeå University, Umeå, Sweden; 10 Department of Women’s and Children’s Health, Karolinska Institutet, Stockholm, Sweden

**Keywords:** Cancer, Young adult, HRQoL, Supportive care needs, Survivors, Psycho-oncology

## Abstract

**Purpose:**

To examine health-related quality of life (HRQoL) and supportive care needs among young adult (YA) cancer survivors up to 3 years post-diagnosis.

**Methods:**

A national cohort of individuals diagnosed at 18–39 years with breast, cervical, ovarian, or testicular cancer, lymphoma or brain tumor was approached with surveys at 1.5 (*n* = 1010, response rate 67%) and 3 (*n* = 722) years post-diagnosis. HRQoL was measured using the EORTC QLQ-C30. Scores were dichotomized using cut-off scores to predict supportive care needs in the Supportive Care Needs Survey-Long Form 59 (SCNS-LF59). Swedish cancer quality registers provided clinical data. Factors predicting need of support at 1.5 and 3 years post-diagnosis were identified using logistic regression.

**Results:**

HRQoL improvements over time were trivial to small. At both time points, a majority of respondents rated HRQoL levels indicating supportive care needs. At 1.5 years post-diagnosis, the risk of having support needs was lower among survivors with testicular cancer (compared to lymphoma) or university-level education, and higher among those on treatment (predominantly endocrine therapy). At 3 years post-diagnosis, when controlling for previous HRQoL scores, most correlations persisted, and poor self-rated household economy and chronic health conditions were additionally associated with supportive care needs.

**Conclusion:**

A majority of YAs diagnosed with cancer rate HRQoL at levels indicating support needs up to 3 years post-diagnosis. Testicular cancer survivors are at lower risk of having support needs. Concurrent health conditions and poor finances are linked to lower HRQoL. More efforts are needed to provide adequate, age-appropriate support to YA cancer survivors.

**Supplementary Information:**

The online version contains supplementary material available at 10.1007/s00520-024-08896-3.

## Background

Of the three million young adults (YAs, here defined as 18–39-year-olds) in Sweden [1], around 2500 are diagnosed with cancer yearly [2]. As cancer incidence and survival rates have risen [3, 4], so has the number of survivors. Increasing attention is being directed at health-related quality of life (HRQoL) in survivorship (starting from cancer diagnosis [5]). HRQoL is considered to be a multidimensional concept representing a person’s perception of how illness and treatment impacts on physical, social, and psychological functioning, and overall health and quality of life [6].

The characteristics of cancer in YAs differ from those in children and older adults [7]. Furthermore, many psychosocial challenges are particularly salient in young adulthood—the time when most people establish themselves regarding education, career, social life, romantic relationships, and family building. Being diagnosed with cancer can interfere with such development. The relative HRQoL impairment in cancer survivors compared to age-matched healthy controls is greater among YAs than in other age groups [8], suggesting that consequences of disease and treatment might be especially impactful in this otherwise relatively healthy group. Studies focusing on HRQoL specifically in YAs are relatively few, and there is a particular paucity of longitudinal studies. In one longitudinal study of adolescent and YA patients with mixed cancer diagnoses, HRQoL deteriorated on treatment, and improved after treatment completion [9], with the most improvement occurring during the first year post-diagnosis. At 2 years post-diagnosis, HRQoL scores were still impaired compared to norm values [9]. In another study on mixed diagnosis YA cancer survivors, improvements in life satisfaction over a 12-month period starting from mixed time points 0–4 years post-diagnosis were seen [10]. Whether HRQoL changes after the first 2 years post-diagnosis remains unclear.

Variables that have been linked to worse HRQoL among YA cancer patients in the literature include the following: female sex [11–13], poor prognosis [9], being on treatment [14, 15], not working or studying [12–14], having a high symptom burden [12–15], comorbid conditions [13], low educational level [15], lack of health insurance [15], being unmarried [15], identifying as Hispanic or Black [15, 16], and having a migration background [12]. Poor HRQoL has been associated with needs of supportive care [17], defined by the National Cancer Institute as physical, psychological, social, or spiritual care aiming to improve the quality of life of people who have an illness or disease [18]. The overall aim of this study was to examine HRQoL and supportive care needs among YA cancer survivors in the first 3 years of survivorship and to identify factors associated with supportive care needs.

## Methods

### Data collection

This study is part of the Fertility and Sexuality following Cancer (Fex-Can) Cohort study [19]. Participants were identified in Swedish cancer quality registers. The inclusion criteria were as follows: being diagnosed with breast, cervical, ovarian, or testicular cancer, lymphoma, or a primary brain tumor, at an age of 18–39 years, between January 2016 and August 2017 (these diagnoses are relatively common among YAs and can affect fertility or sexual function). The eligible were requested to answer surveys when most had completed primary treatment at approximately 1.5 and 3 years post-diagnosis. Exclusion from the first survey (*n* = 36) was due to self-reported cognitive impairment (*n* = 3), death (*n* = 12), invalid postal address (*n* = 18), and administrative failure (*n* = 3). Of 1499 eligible patients, 1010 answered the first survey (67% response rate). Between the first and second survey, 35 participants were excluded due to death (*n* = 28) and invalid postal address (*n* = 7). The second survey was completed by 722 survivors (74% response rate). Study methods and recruitment have been described in detail elsewhere [19].

### Measures

#### Sociodemographic variables from surveys

Participants reported their country of birth at 1.5 years post-diagnosis. At 3 years post-diagnosis, they subjectively rated their household economy (response alternatives: very good/rather good/not particularly good/not good at all/don’t know). Study-specific items about time-sensitive variables (e.g., educational level, relationship status) were included at both time points.

#### HRQoL and supportive care needs

HRQoL was measured using an instrument developed by the European Organisation for Research and Treatment of Cancer (EORTC), the QLQ-C30 [20]. It includes single items and nine multi-item scales. Responses are given on 4-point Likert scales (not at all/a little/quite a bit/very much) for all items except the Global QoL/Health items, which are graded on 7-point scales. Raw scores are transformed into 0–100 scales; higher scores reflect better QoL/functioning and greater symptom burden. To identify patients with supportive care needs, we used cut-off scores developed for YAs with cancer by Lidington et al. [21]. They anchored EORTC QLQ-C30 scores to self-reported needs as measured by corresponding items on the Supportive Care Needs Survey-long form (SCNS-LF59) [22]. The SCNS-LF59 assesses care needs as a function of patients’ concern/discomfort with specific issues and their perceived need of additional help with this as a result of having cancer. For example, the role function scale on the EORTC QLQ-C30 was anchored to having concern or discomfort related to “Not being able to do the things you used to do” on the SCNS-LF59, and perceived need (little/some/strong) for additional help with this as a result of having cancer [21]. Based on this, cut-off scores in EORTC QLQ-C30 scales that reliably predict need for supportive care on the SCNS-LF59 were established [21]. Cut-off scores are available for the following scales: Global Health/QoL, Physical, Role, Social, and Emotional Function, Fatigue, Nausea/Vomiting, Pain, Sleep Disturbances, and Financial Difficulties. All these scales, except Financial Difficulties (since self-reported household economy was used as an independent variable), were used as outcomes in our regression models and will be referred to as “the selected scales.”

#### Clinical variables

Clinical data (e.g., age at diagnosis, sex, and cancer type) were collected from the Swedish cancer quality registers [23–27]. Clinical registry data were used to classify each individual’s treatment according to the ITR-YA [28] as Least intensive/extensive (Level 1), Moderately intensive/extensive (Level 2), Very intensive/extensive (Level 3), or Most intensive/extensive (Level 4).

#### Self-reported disease-related data

Self-reported ongoing cancer treatment at 1.5 and 3 years post-diagnosis included chemotherapy, radiation therapy, endocrine therapy, and other treatments (e.g., immunotherapy) and was dichotomized (on/off treatment). At 3 years post-diagnosis, participants were also asked to answer the open-ended question: “Do you have any chronic disease or health issue (lasting at least 6 months)?” Answers were examined by two of the authors (one with a university degree in medicine and one clinical oncologist), and somatic and psychiatric diseases and disorders were included in the chronic conditions variable. Evident cancer sequelae were excluded.

### Statistical methods

Attrition analyses were performed with chi squared-tests and *t*-tests, comparing those who responded at both time points with those who responded only at 1.5 years (age, sex, cancer type, and ITR). Changes in EORTC QLQ-C30 means between time points were investigated using paired *t*-tests. *P*-values < 0.05 were considered statistically significant. Effect sizes were interpreted based on Cohen’s *d* as small (0.2), medium (0.5), or large (0.8). Proportions of individuals with poor HRQoL scores indicative of supportive care needs at 1.5 and 3 years post-diagnosis were calculated.

Binary logistic regression analyses were performed to identify correlations between independent variables and the outcomes (i.e., poor scores indicating supportive care needs in the selected scales). Separate analyses were performed for the two time points. For time-sensitive, independent variables (e.g., relationship status), variable data from the time point of the outcome was used. The independent variables planned for inclusion in regression models were chosen *à priori* and comprised variables previously linked to HRQoL in the literature, including age, sex (male/female), diagnosis, educational level (ongoing or completed university or college education/other), current treatment status (on/off treatment), occupation (working or studying/other), ITR (level 1–2/level 3–4), country of birth (Sweden/other), and relationship status (partnered/no partner). Parental status (child(ren)/no children) was also included in the regression models. The 3-year post-diagnosis analyses included additional independent variables: self-reported household economic status (very or rather good/not particularly good or not good at all) and chronic health condition(s) (none/ ≥ 1 condition), as well as the scores at 1.5 years post-diagnosis for the respective EORTC domains.

Intervariable correlations were investigated and redundant variables excluded. Sex and intensity of treatment (ITR) were excluded from further analysis as they correlated strongly to diagnosis. Occupation was excluded due to multicollinearity with self-reported household economy. As the vast majority of participants on treatment at 3 years post-diagnosis were breast cancer patients receiving endocrine therapy, the treatment status variable was excluded from the 3-year models. Finally, multivariable logistic regression models were conducted for both time points, with poor HRQoL scores (indicative of supportive care needs) in the selected scales as outcomes.

## Results

The mean age at cancer diagnosis was 32 years. About two-thirds of participants were female (Table 1).

**Table 1:** Sociodemographic and clinical characteristics of young adult cancer survivors at 1.5 and 3 years post-diagnosis

	1.5 years post-diagnosis *n* = 1010^a^ *n* (%)	3 years post-diagnosis *n* = 722^a^ *n* (%)
Sociodemographics		
Country of birth		
Sweden Other	851 (84) 157 (16)	628 (87) 93 (13)
Educational level		
University/college^b^ Other	559 (56) 449 (44)	434 (61) 282 (39)
Occupation		
Working and/or studying Other	799 (79) 209 (21)	632 (88) 83 (12)
Self-reported household economy		
Very or rather good Not that or not at all good	NR NR	602 (85) 107 (15)
Relationship status		
Partner No partner	830 (82) 177 (18)	593 (83) 123 (17)
Parental status		
Child(ren) No children	621 (62) 380 (38)	475 (66) 239 (34)
Clinical characteristics		
Sex		
Female Male	694 (69) 316 (31)	504 (70) 218 (30)
Age at diagnosis		
Mean (SD) 18–29 30–35 36–39	32.4 (5.2) 288 (28) 362 (36) 360 (36)	32.4 (5.2) 208 (29) 255 (35) 259 (36)
Concurrent chronic health conditions		
0 ≥ 1	NR NR	620 (87) 92 (13)
Diagnosis		
Lymphoma Breast cancer Cervical cancer Ovarian cancer Brain tumor Testicular cancer	116 (12) 349 (35) 190 (19) 32 (3) 123 (12) 200 (20)	77 (11) 260 (36) 128 (18) 24 (3) 92 (13) 141 (20)
Intensity of treatment		
1 (least intensive) 2 (moderately intensive) 3 (very intensive) 4 (most intensive)	228 (23) 272 (28) 454 (46) 31 (3)	156 (22) 214 (30) 316 (45) 20 (3)
Current treatment status		
On treatment *Endocrine therapy* *Radiation therapy* *Chemotherapy* *Other* Off treatment	288 (29) *212* *17* *52* *48* 714 (71)	164 (23) *142* *2* *14* *13* 550 (77)

*NR* not reported, *SD* standard deviation

^a^Percentages do not add up due to rounding. Numbers do not add up due to missing values (≤ 10 for all variables except household economy (missing = 13) and intensity of treatment (missing = 25))

^b^Ongoing or completed university or college

The majority of respondents (> 80%) were partnered, and more than half had children. Breast cancer was the most common diagnosis, followed by testicular and cervical cancer. About half had received very or the most intensive/extensive treatment according to the ITR scale. At 3 years post-diagnosis, one in four reported ongoing treatment, with endocrine therapy being the most common treatment (*n* = 142), and breast cancer being the most common diagnosis among those receiving treatment (*n* = 147).

Participation rates at 1.5 years post-diagnosis were significantly lower among males than females, especially younger males, and among females with ovarian cancer and brain tumors compared to remaining diagnoses, as previously reported [29]. Respondents and non-respondents at 1.5 years did not differ significantly regarding cancer treatment intensity according to ITR. Attrition analyses showed no statistically significant differences in age, diagnosis, or intensity of treatment between respondents and non-respondents (by sex) at the 3-year assessment (data not shown).

### Changes in HRQoL over time

**Fig. 1:**
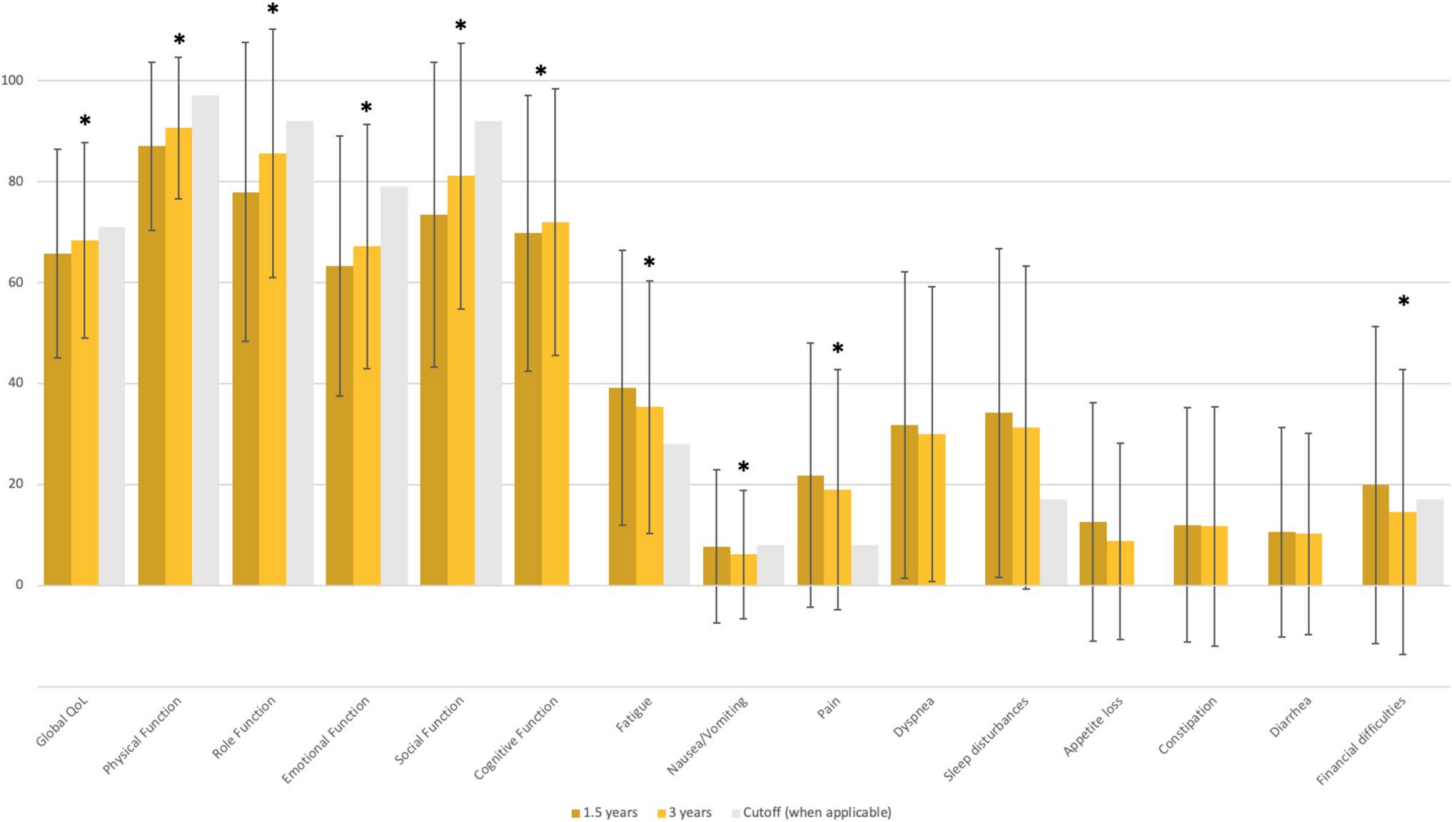
EORTC QLQ-C30 mean scores with standard deviations at 1.5 and 3 years post-diagnosis and cut-off scores indicating supportive care needs. For Global QoL and function scales, higher scores indicate better Global health/QoL or function. For symptom scales, higher scores indicate higher symptom levels. Cut-off scores defined by Lidington et al. [21]. *Two-tailed *P* < 0.05 in paired *t*-tests comparing mean scores at the two time points

Mean values and standard deviations for all EORTC QLQ-C30 scales and single items at 1.5 and 3 years post-diagnosis (by diagnosis) and mean changes over time (for the total sample) are presented in Table 2. Mean scores for the total sample are also presented in Fig. 1. The mean scores in all selected scales except Sleep Disturbances improved over time (range *P* < 0.001–0.047); effect sizes were trivial to small (Cohen’s *d* = 0.08–0.23) (Table 2).

**Table 2:** Mean (SD) values for EORTC QLQ-C30 scales at 1.5 and 3 years post-diagnosis; changes over time with effect sizes

	Scores and changes, all diagnoses			Mean scores by diagnosis					
	Score Mean (SD)	Change Mean (SD)	Effect size Cohen’s d	Lymphoma Mean (SD)	Breast cancer Mean (SD)	Cervical cancer Mean (SD)	Ovarian cancer Mean (SD)	Brain tumor Mean (SD)	Testicular cancer Mean (SD)
Global QoL									
1.5 years 3 years	65.8 (20.7) 68.4 (19.4)	** + 1.5 (19.2)**	0.08	66.2 (19.6)68.6 (17.3)	63.3 (19.6) 66.9 (19.9)	65.5 (22.6) 68.4 (19.9)	56.5 (23.5) 65.9 (15.2)	65.8 (20.5) 68.1 (21.4)	71.8 (19.8) 71.6 (18.3)
Physical Function									
1.5 years 3 years	87.0 (16.7) 90.6 (14.0)	** + 2.1 (13.9)**	0.15	86.0 (17.3)89.6 (13.8)	84.9 (16.9) 89.5 (13.6)	87.9 (17.3) 90.9 (13.8)	81.2 (22.2) 90.6 (13.9)	84.7 (16.5) 87.2 (18.7)	93.1 (12.2) 94.9 (10.0)
Role Function									
1.5 years 3 years	77.9 (29.6) 85.6 (24.6)	** + 6.4 (29.4)**	0.22	79.1 (27.8)84.9 (23.1)	73.5 (30.0) 83.6 (24.5)	81.2 (29.4) 87.0 (25.4)	67.2 (38.8) 86.4 (26.0)	67.5 (32.8) 76.6 (31.6)	89.8 (20.6) 94.0 (16.0)
Emotional Function									
1.5 years 3 years	63.3 (25.8) 67.2 (24.2)	** + 3.4 (23.4)**	0.15	60.9 (24.8)66.4 (24.0)	58.2 (25.3) 65.2 (24.2)	63.2 (27.6) 66.0 (24.0)	56.8 (30.4) 59.1 (24.2)	68.9 (23.4) 67.5 (24.7)	71.3 (23.7) 73.7 (23.6)
Social Function									
1.5 years 3 years	73.5 (30.2) 81.1 (26.3)	** + 6.0 (26.4)**	0.23	75.7 (30.1)81.8 (24.4)	65.0 (31.1) 75.2 (27.9)	77.6 (29.3) 84.4 (26.4)	65.1 (37.7) 87.9 (21.9)	72.4 (29.3) 76.3 (28.9)	85.3 (23.6) 90.9 (18.9)
Cognitive Function									
1.5 years 3 years	69.8 (27.3) 72.0 (26.4)	** + 1.8 (23.2)**	0.08	71.3 (23.4)71.6 (24.5)	63.2 (28.9) 67.6 (27.7)	71.1 (27.1) 73.8 (26.1)	64.6 (29.3) 70.4 (23.0)	71.2 (26.5) 69.3 (26.0)	79.1 (23.5) 80.8 (23.7)
Fatigue									
1.5 years 3 years	39.2 (27.2) 35.4 (25.0)	** − 2.3 (22.7)**	0.10	40.3 (25.8)39.9 (21.0)	44.0 (26.3) 38.4 (26.5)	37.1 (28.4) 31.9 (24.7)	49.1 (29.8) 44.4 (21.7)	42.9 (26.8) 39.3 (26.3)	27.8 (24.4) 26.7 (21.7)
Nausea/Vomiting									
1.5 years 3 years	7.8 (15.1) 6.2 (12.7)	** − 1.3 (16.7)**	0.08	9.3 (17.1)7.4 (13.0)	6.9 (12.6) 8.0 (15.4)	10.2 (17.2) 5.4 (11.1)	15.1 (21.7) 9.8 (13.3)	7.6 (14.1) 5.4 (10.5)	5.2 (14.7) 3.0 (7.9)
Pain									
1.5 years 3 years	21.9 (26.1) 19.0 (23.8)	** − 1.8 (24.0)**	0.08	19.9 (22.1)18.0 (23.4)	28.2 (28.5) 23.3 (25.8)	19.7 (25.6) 18.8 (23.3)	28.6 (32.6) 21.2 (21.3)	19.0 (25.1) 15.9 (21.3)	14.5 (20.4) 13.6 (21.5)
Dyspnea									
1.5 years 3 years	31.8 (30.3) 30.0 (29.2)	** − **1.7 (32.8)	N/A	39.1 (32.2)34.7 (30.7)	33.2 (30.1) 28.8 (29.1)	26.8 (29.7) 29.1 (30.9)	35.4 (33.8) 24.2 (23.4)	30.0 (28.7) 32.6 (30.6)	30.1 (29.7) 29.7 (26.9)
Sleep Disturbances									
1.5 years 3 years	34.2 (32.6) 31.3 (32.0)	** − **0.9 (31.6)	N/A	37.1 (32.7)35.6 (33.3)	41.4 (32.4) 35.8 (33.1)	34.2 (34.2) 27.3 (31.3)	31.2 (30.5) 34.8 (37.8)	30.6 (33.2) 30.7 (32.1)	22.3 (27.3) 24.2 (27.5)
Appetite Loss									
1.5 years 3 years	12.7 (23.6) 8.8 (19.4)	** − 2.0 (23.7)**	0.08	14.3 (22.6)9.9 (17.2)	12.3 (23.4) 9.4 (21.8)	15.2 (26.3) 8.7 (17.5)	20.8 (31.4) 6.1 (16.7)	15.7 (24.7) 11.5 (21.9)	6.9 (18.2) 5.6 (15.4)
Constipation									
1.5 years 3 years	12.0 (23.2) 11.8 (23.7)	0.0 (26.7)	N/A	10.1 (21.7)9.0 (17.7)	14.8 (25.3) 15.0 (26.9)	14.8 (25.5) 13.4 (24.6)	13.5 (22.2) 15.2 (24.6)	13.0 (25.2) 14.1 (26.4)	4.4 (12.7) 3.6 (13.2)
Diarrhea									
1.5 years 3 years	10.6 (20.7) 10.3 (19.9)	+ 0.5 (24.3)	N/A	13.6 (22.9)10.4 (15.5)	8.6 (18.1) 7.2 (17.6)	13.7 (24.2) 15.6 (24.6)	10.4 (23.1) 12.1 (19.4)	9.4 (20.3) 10.7 (20.5)	10.5 (19.4) 10.6 (20.1)
Financial Difficulties									
1.5 years 3 years	20.0 (31.4) 14.6 (28.2)	** − 3.0 (25.7)**	0.12	21.3 (30.4)13.1 (25.2)	26.5 (34.7) 15.6 (29.0)	13.0 (25.6) 10.6 (24.5)	25.0 (36.9) 22.7 (34.7)	22.8 (34.3) 25.2 (35.8)	11.8 (24.2) 9.2 (22.0)

Shown overall and stratified by cancer type. Missing values per diagnosis = 0–6. Missing values for total sample = 13–18. Missing values for change over time (total sample, 17–24). Significant changes over time (two-tailed *P* < 0.05 in paired *t*-tests) in bold

*N/A* not applicable, *QoL* quality of life, *SD* standard deviation

Bold values indicate statistical significance

Among those who were on treatment at 1.5 but not 3 years post-diagnosis (*n* = 64, missing = 1–3), improvements with small to medium effect sizes were observed in several scales (data not shown). Mean scores for most outcomes were significantly worse among females compared to males (Cohen’s *d* = 0.16–0.48), see Supplementary material 1.

### HRQoL scores indicative of supportive care needs

A high prevalence (> 50%) of poor EORTC QLQ-C30 scores indicating supportive care needs at 3 years post-diagnosis was observed in most of the selected scales at both time points (Table 3).

**Table 3:** Number and proportions (%) of young adult cancer survivors with supportive care needs at 1.5 and 3 years post-diagnosis

	Total	Lymphoma	Breast cancer	Cervical cancer	Ovarian cancer	Brain tumor	Testicular cancer
*n* (1.5 years)^a^ *n* (3 years)^a^	1010 722	116 77	349 260	190 128	32 24	123 92	200 141
Scale/item	*n* (%)	*n* (%)	*n* (%)	*n* (%)	*n* (%)	*n* (%)	*n* (%)
Global QoL							
1.5 years 3 years	573 (58) 373 (53)	65 (56) 39 (53)	219 (63) 149 (58)	104 (56) 58 (46)	22 (69) 14 (64)	73 (60) 47 (52)	90 (46) 66 (48)
Physical Function							
1.5 years 3 years	627 (63) 381 (54)	75 (65) 47 (64)	253 (73) 151 (58)	101 (55) 61 (48)	23 (72) 13 (59)	89 (74) 56 (62)	86 (44) 53 (38)
Role Function							
1.5 years 3 years	470 (47) 253 (36)	55 (48) 28 (38)	196 (57) 109 (42)	71 (39) 40 (32)	18 (58) 7 (32)	75 (62) 44 (49)	55 (28) 25 (18)
Emotional Function							
1.5 years 3 years	692 (70) 460 (65)	85 (74) 51 (69)	275 (80) 179 (69)	124 (67) 83 (65)	20 (62) 17 (77)	75 (62) 60 (67)	113 (57) 70 (51)
Social Function							
1.5 years 3 years	568 (57) 330 (46)	59 (52) 38 (51)	246 (71) 154 (60)	91 (49) 46 (36)	19 (59) 7 (32)	78 (64) 48 (53)	75 (38) 37 (27)
Fatigue							
1.5 years 3 years	615 (62) 395 (56)	74 (64) 53 (72)	251 (72) 157 (61)	101 (55) 61 (48)	22 (69) 16 (73)	83 (69) 52 (58)	84 (43) 56 (41)
Nausea/Vomiting							
1.5 years 3 years	292 (29) 184 (26)	37 (32) 24 (32)	104 (30) 78 (30)	68 (37) 30 (24)	15 (47) 10 (46)	34 (28) 22 (24)	34 (17) 20 (14)
Pain							
1.5 years 3 years	575 (58) 379 (54)	68 (59) 35 (47)	234 (67) 159 (62)	99 (54) 67 (53)	19 (59) 14 (64)	60 (50) 44 (49)	95 (48) 60 (44)
Sleep Disturbances							
1.5 years 3 years	633 (64) 417 (59)	77 (67) 49 (66)	260 (75) 165 (64)	113 (61) 66 (52)	20 (62) 12 (54)	68 (56) 53 (59)	95 (48) 72 (52)

Supportive care needs defined according to cut-off scores Lidington et al. [21]

^a^Missing values per diagnosis at 1.5 years = 0–6; at 3 years = 1–4; Missing values for the total sample at 1.5 years = 13–17; at 3 years = 13–14

Overall, the prevalence of supportive care needs decreased over time (statistical significance not calculated), yet was still high at 3 years post-diagnosis, especially for Emotional Function (65%) and Sleep Disturbances (59%). Even among participants who were off treatment, and might be expected to be experiencing less side effects, a majority of scores were poor in most scales (supplementary material 2).

### Factors associated with HRQoL scores indicative of supportive care needs 1.5 years post-diagnosis

In the multivariable logistic regression analyses, being on treatment was correlated to poor scores at 1.5 years post-diagnosis for Global Health/QoL, Physical, Role, and Social Function, as well as Fatigue (Table 4). Compared to those with lymphoma, participants with testicular cancer were less likely to have supportive care needs in all selected scales except Global Health/QoL and those with brain tumor had a lower risk of supportive care needs in the Emotional Function scale. Low educational level was associated with supportive care needs regarding Global Health/QoL, Physical and Social Function, and Fatigue. Not having a partner was associated with Role Function scores indicative of supportive care needs. There was a lower risk of supportive care needs regarding Pain among those who were parents. Risk of supportive care needs regarding Social Function increased with age. Country of birth was not associated with any of the outcomes.

**Table 4:** Factors predictive of supportive care needs in young adult cancer survivors at 1.5 years post-diagnosis in multivariable logistic regression models

Factors	Global QoL OR (CI)	Physical Function OR (CI)	Role Function OR (CI)	Emotional Function OR (CI)	Social Function OR (CI)	Fatigue OR (CI)	Nausea/Vomiting OR (CI)	Pain OR (CI)	Sleep Disturbances OR (CI)
Age									
	1.00 (0.97–1.03)	1.00 (0.96–1.03)	1.00 (0.97–1.03)	1.00 (0.97–1.04)	**1.03 (1.00–1.07)**^**a**^	0.99 (0.96–1.02)	0.97 (0.94–1.00)	1.02 (0.99–1.05)	1.00 (0.96–1.03)
Cancer type									
Lymphoma (ref)									
Breast	1.18 (0.71–1.95)	1.10 (0.64–1.87)	1.01 (0.61–1.67)	1.29 (0.73–2.28)	1.41 (0.84–2.36)	1.16 (0.69–1.96)	0.87 (0.50–1.49)	1.25 (0.75–2.08)	1.25 (0.74–2.13)
Cervical	1.05 (0.64–1.70)	0.71 (0.43–1.18)	0.76 (0.47–1.24)	0.75 (0.44–1.27)	0.85 (0.53–1.38)	0.73 (0.45–1.20)	1.34 (0.81–2.23)	0.81 (0.50–1.31)	0.82 (0.50–1.35)
Ovarian	1.79 (0.75–4.27)	1.42 (0.57–3.51)	1.58 (0.69–3.61)	0.64 (0.27–1.50)	1.40 (0.61–3.19)	1.19 (0.51–2.79)	2.08 (0.92–4.70)	1.05 (0.46–2.39)	0.75 (0.33–1.71)
Brain tumor	1.11 (0.66–1.88)	1.40 (0.79–2.48)	1.67 (0.99–2.83)	**0.57 (0.32–1.00)**^**a**^	1.52 (0.89–2.59)	1.17 (0.68–2.03)	0.80 (0.46–1.41)	0.66 (0.39–1.11)	0.60 (0.35–1.02)
Testicular	0.67 (0.42–1.07)	**0.40 (0.24–0.65)**^**c**^	**0.44 (0.27–0.71)**^**c**^	**0.49 (0.29–0.81)**^**b**^	**0.56 (0.34–0.90)**^**a**^	**0.44 (0.27–0.71)**^**c**^	**0.45 (0.26–0.78)**^**b**^	**0.62 (0.38–1.00)**^**a**^	**0.46 (0.28–0.76)**^**b**^
On/off treatment									
Off (ref)									
On	**1.46 (1.01–2.11)**^**a**^	**2.05 (1.37–3.07)**^**c**^	**1.76 (1.22–2.53)**^**b**^	1.19 (0.79–1.80)	**1.95 (1.32–2.86)**^**c**^	**1.80 (1.21–2.66)**^**b**^	1.42 (0.96–2.09)	1.31 (0.90–1.89)	1.46 (0.99–2.16)
Relationship status									
Partner (ref)									
No partner	1.27 (0.88–1.84)	1.11 (0.75–1.65)	**1.50 (1.04–2.19)**^**a**^	0.75 (0.51–1.11)	0.78 (0.54–1.14)	0.91 (0.62–1.33)	0.90 (0.61–1.35)	0.75 (0.52–1.08)	1.18 (0.81–1.74)
Have child(ren)									
No (ref)									
Yes	0.94 (0.67–1.31)	0.78 (0.55–1.11)	1.23 (0.87–1.72)	0.77 (0.54–1.10)	0.90 (0.64–1.27)	0.98 (0.70–1.38)	1.04 (0.73–1.50)	**0.67 (0.48–0.94)**^**a**^	0.92 (0.65–1.29)
Country of birth									
Sweden (ref)									
Other	0.85 (0.59–1.22)	1.48 (0.99–2.22)	1.07 (0.74–1.55)	1.15 (0.77–1.73)	0.90 (0.62–1.31)	1.13 (0.77–1.66)	1.31 (0.90–1.91)	1.22 (0.85–1.77)	1.20 (0.82–1.76)
Education									
Other (ref)									
University^d^	**0.68 (0.52–0.89)**^**b**^	**0.56 (0.42–0.74)**^**c**^	0.80 (0.61–1.04)	0.89 (0.66–1.18)	**0.70 (0.54–0.93)**^**a**^	**0.70 (0.53–0.93)**^**a**^	0.89 (0.66–1.18)	0.79 (0.60–1.03)	0.88 (0.66–1.16)

Supportive care needs based on EORTC QLQ-C30 cut-off scores Lidington et al. [21]. Age as a continuous variable. Significant values (*P* < 0.05) in bold. Missing values = 30–34

*CI* confidence interval, *OR* odds ratio, *QoL* quality of life

^a^*P* < 0.05

^b^*P* < 0.01

^c^*P* < 0.001

^d^Ongoing or completed university or college

Bold values indicate statistical significance

### Factors associated with HRQoL scores indicative of supportive care needs 3 years post-diagnosis

In the multivariable regression analyses, favorable HRQoL scores at 1.5 years post-diagnosis were, as expected, associated with less supportive care needs at 3 years post-diagnosis in all selected scales (Table 5).

**Table 5:** Factors predictive of supportive care needs in young adult cancer survivors at 3 years post-diagnosis in multivariable logistic regression models

Factors	Global QoL OR (CI)	Physical Function OR (CI)	Role Function OR (CI)	Emotional Function OR (CI)	Social Function OR (CI)	Fatigue OR (CI)	Nausea/Vomiting OR (CI)	Pain OR (CI)	Sleep Disturbances OR (CI)
Age									
	0.99 (0.95–1.03)	0.99 (0.96–1.03)	1.00 (0.96–1.05)	1.00 (0.96–1.04)	1.02 (0.98–1.07)	1.02 (0.97–1.06)	0.96 (0.92–1.01)	1.02 (0.98–1.06)	1.03 (0.99–1.08)
Cancer type									
Lymphoma (ref)									
Breast	1.26 (0.67–2.36)	0.94 (0.50–1.78)	1.39 (0.73–2.64)	1.20 (0.61–2.34)	1.07 (0.56–2.02)	0.51 (0.25–1.03)	1.16 (0.62–2.17)	**2.07 (1.12–3.85)**^**a**^	0.77 (0.41–1.47)
Cervical	0.79 (0.40–1.55)	0.72 (0.37–1.40)	0.97 (0.48–1.95)	1.26 (0.62–2.58)	0.58 (0.29–1.16)	**0.45 (0.21–0.94)**^**a**^	0.64 (0.32–1.28)	1.84 (0.96–3.56)	0.60 (0.30–1.19)
Ovarian	1.16 (0.36–3.67)	0.56 (0.18–1.77)	0.43 (0.13–1.46)	1.36 (0.37–5.00)	**0.15 (0.04–0.58)**^**b**^	0.67 (0.18–2.47)	1.47 (0.51–4.27)	1.79 (0.59–5.46)	0.46 (0.15–1.42)
Brain tumor	1.01 (0.49–2.08)	0.85 (0.41–1.76)	1.60 (0.78–3.30)	1.19 (0.56–2.54)	1.07 (0.52–2.21)	0.46 (0.21–1.01)	0.65 (0.31–1.36)	1.15 (0.57–2.34)	0.94 (0.45–1.95)
Testicular	1.10 (0.57–2.12)	**0.49 (0.25–0.95)**^**a**^	0.48 (0.23–1.01)	0.61 (0.30–1.21)	**0.41 (0.20–0.82)**^**a**^	**0.35 (0.17–0.73)**^**b**^	**0.39 (0.19–0.81)**^**a**^	1.16 (0.61–2.21)	0.84 (0.43–1.64)
Relationship status									
Partner (ref)									
No partner	0.75 (0.45–1.27)	0.73 (0.44–1.22)	0.81 (0.47–1.40)	0.74 (0.43–1.28)	0.81 (0.47–1.39)	0.62 (0.36–1.06)	0.77 (0.45–1.32)	1.41 (0.84–2.36)	1.08 (0.63–1.83)
Self-rated economy									
Good (ref)									
Poor	**2.39 (1.34–4.27)**^**b**^	**2.23 (1.28–3.89)**^**b**^	**3.02 (1.78–5.13)**^**c**^	**2.93 (1.45–5.93)**^**b**^	**2.45 (1.40–4.28)**^**b**^	**2.59 (1.40–4.81)**^**b**^	**2.81 (1.72–4.59)**^**c**^	**1.78 (1.04–3.06)**^**a**^	1.46 (0.84–2.52)
Have child(ren)									
No (ref)									
Yes	1.10 (0.70–1.73)	0.67 (0.43–1.05)	0.98 (0.61–1.57)	**0.49 (0.30–0.79)**^**b**^	1.01 (0.64–1.60)	0.92 (0.57–1.49)	1.20 (0.76–1.92)	**0.60 (0.39–0.93)**^**a**^	0.74 (0.47–1.16)
Country of birth									
Sweden (ref)									
Other	1.10 (0.65–1.87)	1.46 (0.86–2.48)	1.02 (0.59–1.75)	1.16 (0.66–2.02)	0.98 (0.57–1.69)	0.93 (0.53–1.62)	1.07 (0.62–1.83)	1.57 (0.92–2.68)	0.88 (0.52–1.49)
Education									
Other (ref)									
University^d^	0.88 (0.61–1.26)	0.72 (0.50–1.02)	0.85 (0.59–1.24)	**0.60 (0.40–0.88)**^**b**^	**0.69 (0.47–1.00)**^**a**^	0.79 (0.53–1.16)	0.93 (0.64–1.36)	0.78 (0.54–1.11)	0.95 (0.67–1.36)
Chronic condition(s)									
None (ref)									
One or several	**3.17 (1.78–5.65)**^**c**^	**1.76 (1.04–3.00)**^**a**^	**2.32 (1.38–3.90)**^**b**^	**2.09 (1.16–3.77)**^**a**^	**2.04 (1.18–3.52)**^**a**^	1.62 (0.88–2.98)	1.40 (0.84–2.32)	**1.91 (1.11–3.29)**^**a**^	1.04 (0.62–1.76)
Score at 1.5 years									
	**0.94 (0.93–0.96)**^**c**^	**0.93 (0.91–0.95)**^**c**^	**0.97 (0.97–0.98)**^**c**^	**0.96 (0.95–0.97)**^**c**^	**0.96 (0.96–0.97)**^**c**^	**1.05 (1.04–1.07)**^**c**^	**1.03 (1.01–1.04)**^**c**^	**1.04 (1.03–1.05)**^**c**^	**1.04 (1.03–1.04)**^**c**^

Supportive care needs based on EORTC QLQ-C30 cut-off scores Lidington et al. [21]. Age and corresponding EORTC scale scores at 1.5 years post-diagnosis as continuous variables. Significant values (*P* < 0.05) in bold. Missing values = 35–39

*CI* confidence interval, *OR* odds ratio, *QoL* quality of life

^a^*P* < 0.05

^b^*P* < 0.01

^c^*P* < 0.001

^d^Ongoing or completed university or college

Bold values indicate statistical significance

The following factors were significantly associated with care needs at 3 years when controlling for HRQoL scores at 1.5 years post-diagnosis: Having ≥ 1 concurrent health condition (Global Health/QoL, Physical, Role, Emotional, and Social Function, Pain), poor self-reported household economy (all scales except Sleep Disturbances), lower educational level (Emotional and Social Function), and not having children (Emotional Function and Pain). Compared to those with lymphoma, testicular cancer patients were less likely to have supportive care needs regarding Physical and Social Function and Nausea/Vomiting, and ovarian cancer patients were less likely to have supportive care needs regarding Social Function. Breast cancer patients, on the other hand, had a higher risk of supportive care needs (Pain). Among patients with lymphoma, a greater proportion had Fatigue levels indicating support care needs compared to testicular and cervical cancer patients. The remaining factors were not associated with any outcomes. Ongoing treatment was not included in the regression model at 3 years post-diagnosis, as participants on treatment for other diagnoses than breast cancer were few. However, descriptively, ongoing treatment was related to higher proportions of supportive care needs in most scales, with higher treatment-related discrepancies in non-breast cancer participants; see Supplementary material 2.

## Discussion

We examined HRQoL in a national cohort of YA cancer survivors. The inclusion of gynecological cancer and brain tumors in a longitudinal study on HRQoL in YAs is, to our knowledge, novel. Despite statistically significant improvements in HRQoL within the first years of survivorship, a majority of participants had scores indicating supportive care needs related to global QoL, physical, and emotional function, fatigue, pain, and sleep disturbances at 3 years post-diagnosis. Survivors with ≥ 1 concurrent chronic health condition or poor household economy were more likely to rate poor HRQoL. Testicular cancer survivors were less likely to rate poor HRQoL than lymphoma survivors. At 1.5 years post-diagnosis, being on treatment was associated with supportive care needs. At 3 years post-diagnosis, ongoing treatment was descriptively correlated to higher rates of supportive care needs (Supplementary material 2), and this difference was particularly striking among those with other diagnoses than breast cancer.

The trivial to small improvements in EORTC QLQ-C30 scale means between 1.5 and 3 years post-diagnosis are in line with previous findings [9, 10]. As changes reflect the entire sample, slight net improvements might reflect clinically significant improvements among subgroups of participants. It is, however, clear that many participants have persistently poor scores, as poor scores were highly prevalent (> 50% in a majority of the selected scales) at both time points. Previous research has shown poorer HRQoL among YA cancer survivors than in the YA general population [11–13, 15, 30], although levels equal to or exceeding those of the general population > 1 year post-treatment have been observed among lymphoma and testicular cancer survivors [31, 32]. The high prevalence of supportive care needs in our cohort of young adults adds to previous findings in this population [33].

Several clinical factors were found to be related to HRQoL. Being on (adjuvant or relapse) treatment was associated with increased risk for poor scores at 1.5 years post-diagnosis. This result is expected due to previous findings [9] and considering the risk of side-effects. Descriptively, the prevalence of poor scores in all scales at 3 years post-diagnosis was higher among those on treatment, yet also substantial among those off treatment (Supplementary material 2). That differences by treatment status seem greater among those with other diagnoses than breast cancer might be related to disease progression/relapse (as opposed to standard adjuvant treatment). The association between concurrent chronic conditions and poor scores at 3 years post-diagnosis is in line with previous research [13].

Compared to lymphoma survivors, testicular cancer survivors were at lower risk of having supportive care needs at both time points. Testicular cancer has high survival rates, and treatment regimens for early-stage disease are not intensive/extensive [34], which could explain the relatively positive results in this group. Our findings of more supportive care needs regarding fatigue among lymphoma patients corroborate previous results describing fatigue among lymphoma survivors [13, 35].

Poor HRQoL scores indicating supportive care needs among YA cancer survivors were associated with several sociodemographic variables. Participants with university-level education were at lower risk of poor scores in the global, physical, and social function and fatigue scales at 1.5 years post-diagnosis. Poor self-reported household economy was associated with poor HRQoL at 3 years post-diagnosis, in concordance with previous findings in cancer survivors [15]. Survivors with impaired functioning might not be able to work or study to the same extent as before and subsequently lose income. Not working or studying might impact health perception, and/or lead to loss of purpose or community. The public health care system in Sweden has a good coverage and is almost completely tax-funded, yet small cost barriers to appropriate care (e.g., health care appointments) might still impact the HRQoL of cancer survivors. Young adults who are not yet financially stable are likely to face considerable pressure if they cannot work due to illness.

Somewhat surprisingly, partner status was not correlated to poor scores in any of the scales (except for role function at 1.5 years post-diagnosis). This stands in contrast to previous findings, where being in a relationship predicted life satisfaction among cancer survivors [10]. Parenthood was associated with lower risk of poor emotional function and pain scores at 3 years post-diagnosis. It might be that participants with a stable financial situation, better perceived health, or stronger social support networks are more willing to have children, or children might distract their parents from negative emotions and sensations.

Our study identified participants in national cancer quality registers, which provide excellent nationwide coverage and reliable clinical data [36]. Although we reached good response rates overall, participation rates were higher among females—a pattern which is also present in similar studies [37]. Those with high educational level were more likely to respond. Furthermore, our sample’s proportion of people born outside of Sweden was lower than in the Swedish general population [38], possibly due to language barriers (the surveys were in Swedish). Some of the survivors in our cohort also took part in the embedded Fex-Can intervention randomized controlled trial (*n* = 124, intervention group *n* = 64) [39]. As the primary outcomes of the intervention were sexual satisfaction and fertility distress, we deemed the risk that the intervention would influence the results of this study as low and included all cohort participants. Published results from the randomized controlled trial [40], as well as data awaiting to be published, showed no statistically significant differences in EORTC QLQ-C30 summary scores between groups. The use of the well-established EORTC QLQ-C30 strengthens our study; however, this measure fails to address some YA-specific issues, for example fertility distress. Current treatment status was based on self-report (since information on this was lacking in some of the quality registers). While we did not base our assessment of chronic health conditions on a standardized measure, our classification of participants’ self-reports was conducted systematically by a clinical oncologist and a medical school graduate. Due to the number of variables in our models, the power of our results was reduced, possibly hiding associations, especially in smaller subgroups such as ovarian cancer patients. On the other hand, these models allowed us to present a holistic picture including several relevant factors.

It should be noted that when dichotomizing the EORTC QLQ-C30 outcomes using the cut-off scores by Lidington et al. [21] to identify those in need of support, survivors with varying degrees of need from “little” to “strong” are included. The presence of supportive care needs does not automatically imply that the survivor in question would be motivated to participate in rehabilitation [41]. However, it is important to identify survivors who might benefit from support. An evaluation of the Swedish supportive care strategies showed lacking implementation of strategies described in policy and legislation [42]. For example, according to the Swedish national cancer strategy, every patient should have access to a contact nurse, yet only 53% of patients reported having had this. Only a minority of patients reported having a written care plan or having received information about patient advocacy groups. In a recent survey [43] conducted by Ung Cancer, a Swedish advocacy group for cancer survivors aged 16–30, members reported a multitude of needs, including needs for information, physical rehabilitation, and meeting other young cancer survivors. Of those who had received services to meet such needs, a large proportion of respondents did not find the support helpful. One common reason for not taking part of offered support was that it was not perceived as tailored to the YA perspective.

In conclusion, the majority of YA cancer survivors report HRQoL levels indicative of supportive care needs up to 3 years post-diagnosis, with small improvements over time. There are widespread needs for support in this group, particularly among those with financial issues and concurrent chronic health conditions. YAs diagnosed with cancer should be offered follow-up care to identify needs and adequate support should be offered.

## Supplementary Information

Below is the link to the electronic.

Supplementary file1 (DOCX 23 KB)

Supplementary file2 (DOCX 20.3 KB)

## Data Availability

Data is available, upon reasonable request, by contacting the corresponding author.
