# Supplementary material for: Health-related quality of life and supportive care needs in young adult cancer survivors—a longitudinal population-based study
Source: Support Care Cancer. 2024 Oct 22;32(11):742. doi: 10.1007/s00520-024-08896-3 (PMC11496321; doi:10.1007/s00520-024-08896-3)
Supplement: Supplementary file 1 — Supplementary file1 (DOCX 23 KB) [file 520_2024_8896_MOESM1_ESM.docx]

**Health-related quality of life and supportive care needs in young adult cancer survivors - a longitudinal population-based study**

Alexandra Wide, Johan Ahlgren, Karin E. Smedby, Kristina Hellman, Roger Henriksson, Olof Ståhl, Claudia Lampic^*^, and Lena Wettergren^*^

^*^Joint last authorship

**Corresponding author**

Lena Wettergren, Department of Public Health and Caring Sciences, Uppsala University, BMC, Box 564, 751 22 Uppsala, Sweden; [lena.wettergren@.uu.se](mailto:lena.wettergren@.uu.se)

For submission to Supportive Care in Cancer.

**SUPPLEMENTARY MATERIAL 1**

EORTC QLQ-C30 scale scores at 1.5 and 3 years post-diagnosis

|  | **Mean (SD)**  **Total** | **Mean (SD)**  **Female** | **Mean (SD)**  **Male** | **p-value^a^** | **Cohen’s d^b^** |
| --- | --- | --- | --- | --- | --- |
| Global QoL | |  |  |  |  |
| 1.5 years  3 years | 65.8 (20.7)  68.4 (19.4) | 63.6 (20.8)  66.9 (19.6) | 70.8 (19.6)  71.8 (18.5) | **<0.001**  **0.002** | 0.36 (0.22-0.49)  0.26 (0.09-0.42) |
| Physical Function | |  |  |  |  |
| 1.5 years  3 years | 87.0 (16.7) 90.6 (14.0) | 85.3 (17.4)  89.4 (14.4) | 91.0 (14.1)  93.2 (12.6) | **<0.001**  **<0.001** | 0.35 (0.21-0.48)  0.27 (0.11-0.43) |
| Role Function | |  |  |  |  |
| 1.5 years  3 years | 77.9 (29.6)  85.6 (24.6) | 74.9 (30.7)  83.6 (25.8) | 84.4 (25.8)  90.2 (21.0) | **<0.001**  **<0.001** | 0.32 (0.19-0.46)  0.27 (0.11-0.43) |
| Emotional Function | |  |  |  |  |
| 1.5 years  3 years | 63.3 (25.8)  67.2 (24.2) | 59.5 (26.0)  64.8 (24.0) | 71.6 (23.3)  73.0 (23.9) | **<0.001**  **<0.001** | 0.48 (0.34-0.62)  0.34 (0.18-0.51) |
| Social Function | |  |  |  |  |
| 1.5 years  3 years | 73.5 (30.2)  81.1 (26.3) | 69.3 (31.3)  78.1 (27.5) | 82.7 (25.4)  88.3 (21.8) | **<0.001**  **<0.001** | 0.45 (0.32-0.59)  0.39 (0.23-0.56) |
| Cognitive Function | |  |  |  |  |
| 1.5 years  3 years | 69.8 (27.3)  72.0 (26.4) | 66.1 (28.0)  69.1 (26.7) | 77.8 (23.6)  78.7 (24.4) | **<0.001**  **<0.001** | 0.44 (0.30-0.57)  0.37 (0.21-0.53) |
| Fatigue |  |  |  |  |  |
| 1.5 years  3 years | 39.2 (27.2)  35.4 (25.0) | 42.7 (27.2)  38.1 (25.7) | 31.4 (25.3)  29.2 (22.2) | **<0.001**  **<0.001** | 0.42 (0.29-0.56)  0.36 (0.20-0.52) |
| Nausea/Vomiting | |  |  |  |  |
| 1.5 years  3 years | 7.8 (15.1)  6.2 (12.7) | 8.7 (15.6)  7.4 (13.9) | 5.9 (13.8)  3.5 (8.7) | **0.005**  **<0.001** | 0.18 (0.05-0.32)  0.31 (0.14-0.47) |
| Pain |  |  |  |  |  |
| 1.5 years  3 years | 21.9 (26.1)  19.0 (23.8) | 24.6 (27.6)  21.3 (24.6) | 15.8 (21.2)  13.8 (21.1) | **<0.001**  **<0.001** | 0.34 (0.21-0.48)  0.31 (0.15-0.48) |
| Dyspnea |  |  |  |  |  |
| 1.5 years  3 years | 31.8 (30.3)  30.0 (29.2) | 31.5 (30.0)  29.4 (29.7) | 32.4 (31.0)  31.3 (28.0) | 0.685  0.439 | N/A  N/A |
| Sleep Disturbances | |  |  |  |  |
| 1.5 years  3 years | 34.2 (32.6)  31.3 (32.0) | 38.3 (33.2)  33.5 (32.9) | 25.1 (29.2)  26.3 (29.3) | **<0.001**  **0.006** | 0.41 (0.28-0.55)  0.23 (0.06-0.39) |
| Appetite loss | |  |  |  |  |
| 1.5 years  3 years | 12.7 (23.6)  8.8 (19.4) | 14.0 (24.8)  9.7 (20.1) | 9.9 (20.5)  6.6 (17.5) | **0.007**  **0.044** | 0.17 (0.04-0.31)  0.16 (0.00-0.32) |
| Constipation | |  |  |  |  |
| 1.5 years  3 years | 12.0 (23.2)  11.8 (23.7) | 14.4 (25.0)  14.6 (25.8) | 6.5 (17.5)  5.0 (16.1) | **<0.001**  **<0.001** | 0.34 (0.21-0.48)  0.41 (0.25-0.57) |
| Diarrhea |  |  |  |  |  |
| 1.5 years  3 years | 10.6 (20.7)  10.3 (19.9) | 10.5 (20.8)  10.1 (20.2) | 10.9 (20.4)  10.7 (19.2) | 0.757  0.727 | N/A  N/A |
| Financial Difficulties | |  |  |  |  |
| 1.5 years  3 years | 20.0 (31.4)  14.6 (28.2) | 22.2 (32.6)  15.8 (29.1) | 15.1 (27.9)  12.0 (25.6) | **<0.001**  0.083 | 0.23 (0.09-0.36)  N/A |

NOTE: Shown overall and stratified by sex. Missing values=13-18.

Abbreviations: N/A, not applicable; SD, standard deviation.

^a^ Two-tailed p-values (significance threshold <0.05, significant values in bold) for independent samples t-test investigating differences between score means among male and female participants.

^b^Effect sizes (95% confidence intervals) for statistically significant mean differences between the sexes.
