# Supplementary material for: Health-related quality of life and supportive care needs in young adult cancer survivors—a longitudinal population-based study
Source: Support Care Cancer. 2024 Oct 22;32(11):742. doi: 10.1007/s00520-024-08896-3 (PMC11496321; doi:10.1007/s00520-024-08896-3)
Supplement: Supplementary file 2 — Supplementary file2 (DOCX 20.3 KB) [file 520_2024_8896_MOESM2_ESM.docx]

**Health-related quality of life and supportive care needs in young adult cancer survivors - a longitudinal population-based study**

Alexandra Wide, Johan Ahlgren, Karin E. Smedby, Kristina Hellman, Roger Henriksson, Olof Ståhl, Claudia Lampic^*^, and Lena Wettergren^*^

^*^Joint last authorship

**Corresponding author**

Lena Wettergren, Department of Public Health and Caring Sciences, Uppsala University, BMC, Box 564, 751 22 Uppsala, Sweden; [lena.wettergren@uu.se](mailto:lena.wettergren@uu.se)

For submission to Supportive Care in Cancer

**SUPPLEMENTARY MATERIAL 2**

Number and proportions of participants with supportive care needs at 1.5 and 3 years post-diagnosis by treatment status

|  | Total | | Breast cancer | | Other diagnoses | |
| --- | --- | --- | --- | --- | --- | --- |
| Scale/item | **Off treatment**  n=714/543  **n (%)** | **On treatment**  n=288/163  **n (%)** | **Off treatment**  n =114/111  **n (%)** | **On treatment**  n=232/146  **n (%)** | **Off treatment**  n=600/439  **n (%)** | **On treatment**  n=56/17  **n (%)** |
| Global QoL  1.5 years  3 years | 380 (54)  271 (50) | 190 (67)  100 (61) | 71 (62)  59 (53) | 147 (64)  89 (61) | 309 (52)  212 (49) | 43 (78)  11 (65) |
| Physical Function  1.5 years  3 years | 398 (56)  280 (52) | 222 (78)  99 (61) | 74 (65)  63 (57) | 176 (76)  87 (60) | 324 (55)  217 (50) | 46 (84)  12 (71) |
| Role Function  1.5 years  3 years | 292 (42)  178 (33) | 175 (62)  73 (45) | 61 (54)  45 (40) | ¨¨  133 (58)  63 (43) | 231 (39)  133 (31) | 42 (76)  10 (59) |
| Emotional Function  1.5 years  3 years | 464 (66)  341 (63) | 223 (78)  117 (72) | 86 (76)  74 (67) | 186 (81)  104 (71) | 378 (64)  267 (62) | 37 (67)  13 (76) |
| Social Function  1.5 years  3 years | 352 (50)  232 (43) | 213 (75)  96 (59) | 76 (67)  65 (59) | 168 (73)  88 (60) | 276 (47)  167 (39) | 45 (82)  8 (47) |
| Fatigue  1.5 years  3 years | 394 (56)  287 (53) | 217 (76)  106 (65) | 77 (68)  63 (57) | 172 (75)  93 (64) | 317 (54)  224 (52) | 45 (82)  13 (76) |
| Nausea/Vomiting  1.5 years  3 years | 195 (28)  130 (24) | 95 (33)  53 (32) | 34 (30)  30 (27) | 70 (30)  48 (33) | 161 (27)  100 (23) | 25 (46)  5 (29) |
| Pain  1.5 years  3 years | 377 (54)  276 (51) | 192 (67)  101 (62) | 79 (69)  65 (59) | 153 (66)  93 (64) | 298 (50)  211 (49) | 39 (71)  8 (47) |
| Sleep Disturbances  1.5 years  3 years | 413 (59)  305 (56) | 214 (75)  110 (68) | 78 (68)  66 (60) | 179 (78)  99 (68) | 335 (57)  239 (55) | 35 (64)  11 (65 |

NOTE: Presented overall and stratified by breast cancer/other diagnoses. n=number of participants off treatment, shown for 1.5/3 years post-diagnosis. Missing=20-24 at 1.5 years post-diagnosis from total sample (n=1010) and 16-17 at 3 years post-diagnosis from total sample (n=722).

Abbreviations: QoL, Quality of life.
